# Characteristics of elderly diabetes patients: focus on clinical manifestation, pathogenic mechanism, and the role of traditional Chinese medicine

**DOI:** 10.3389/fphar.2023.1339744

**Published:** 2024-01-11

**Authors:** Xiaofei Yang, Chongxiang Xue, Keyu Chen, Dongyang Gao, Han Wang, Cheng Tang

**Affiliations:** ^1^ Beijing University of Chinese Medicine, Beijing, China; ^2^ Institute of Metabolic Diseases, Guang’anmen Hospital, China Academy of Chinese Medical Sciences, Beijing, China

**Keywords:** elderly diabetes, traditional Chinese medicine, clinical manifestation, pathogenic mechanism, treatment

## Abstract

Diabetes mellitus has become a major public health issue globally, putting an enormous burden on global health systems and people. Among all diseased groups, a considerable part of patients are elderly, while their clinical features, pathogenic processes, and medication regimens are different from patients of other ages. Despite the availability of multiple therapies and techniques, there are still numerous elderly diabetes patients suffering from poor blood glucose control, severe complications, and drug adverse effects, which negatively affect the quality of life in their golden years. Traditional Chinese Medicine (TCM) has been widely used in the treatment of diabetes for several decades, and its relevant clinical practice has confirmed that it has a satisfactory effect on alleviating clinical symptoms and mitigating the progression of complications. Chinese herbal medicine and its active components were used widely with obvious clinical advantages by multiple targets and signaling pathways. However, due to the particular features of elderly diabetes, few studies were conducted to explore Traditional Chinese Medicine intervention on elderly diabetic patients. This study reviews the research on clinical features, pathogenic processes, treatment principles, and TCM treatments, hoping to provide fresh perspectives on the prevention and management strategies for elderly diabetes.

## 1 Introduction

Diabetes mellitus (DM) is characterized by an absolute or relative lack of insulin creation as well as insulin resistance (IR) in target tissues, which leads to hyperglycemia and glucose intolerance (Ma et al., 2022). It can be classified into four types: type 1 diabetes (T1DM), type 2 diabetes (T2DM), gestational diabetes, and diabetes driven by islet disease or medicines (Fan et al., 2022; Yang and Yang, 2022). With an expanding lifespan and conversion in diet structure, the number of patients with diabetes-related high-risk factors increases, which contributes to the increased prevalence of diabetes, especially among elderly people (LeRoith et al., 2019). Elderly diabetes refers to the patients above the age of sixty, including patients diagnosed with diabetes before the age of sixty and patients diagnosed with diabetes after the age of sixty (Chinese Elderly Type 2 Diabetes Prevention and Treatment of Clinical Guidelines Writing Group et al., 2022). According to prior studies, around 28.8% of Chinese individuals aged 60–69 suffered from diabetes. This figure jumped to 31.8% among those aged over 70, which was significantly higher than young and middle-aged populations (Li et al., 2020). However, the present knowledge, diagnosis, treatment, and self-management capacity of elderly diabetes patients is inadequate, which causes the blood glucose level out of control (Chinese Elderly Type 2 Diabetes Prevention and Treatment of Clinical Guidelines Writing Group et al., 2022). Many people were first diagnosed with diabetes because of serious complications such as ischemic cardiovascular and cerebrovascular disease. This not only greatly affects patients' quality of life, but also creates an enormous burden on society and the medical system.

Current treatments for diabetes include insulin secretagogues, biguanides, insulin sensitizers, alpha-glucosidase inhibitors, incretin mimetics, amylin antagonists, and sodium-glucose co-transporter-2 (SGLT2) inhibitors, glucagon-like peptide-1 (GLP-1) receptors agonists (Padhi et al., 2020). Though these drugs can enhance insulin sensitivity, promote insulin secretion, and improve metabolic disorders, there are still a large number of patients suffering from poor blood glucose control, serious complications and drug side effects. Previous studies have shown that Traditional Chinese Medicine (TCM) was extremely effective in stabilizing blood glucose fluctuations, preventing the occurrence of brittle diabetes, slowing the development of complications, and enhancing patients’ life quality (Tian et al., 2019). Therefore, attention has steadily been drawn to comprehensive regimens with integrative medicine for diabetes.

Pathogenic pathways peculiar to elderly diabetes include gut microbiota disturbance, cell senescence, mitochondrial failure, oxidative stress, and alterations in epigenetic expression. Also, many Chinese herbs (such as *Puerariae Lobatae, Radix Scutellaria baicalensis Georgi*, *Salvia miltiorrhiza*) play a role in elderly diabetes through the aforementioned mechanisms (Xiao et al., 2020; Wang et al., 2022c; Yingrui et al., 2022; Niu et al., 2023). Furthermore, different Chinese medicine formulations may be chosen for symptomatic therapy based on the stage of diabetes to fully exert the advantage of TCM. Here, we reviewed the characteristics, pathogenesis and related TCM treatments of elderly diabetes, and analyzed the research status and potential clinical situations.

## 2 Clinical features in elderly diabetes

### 2.1 Invisible typical symptoms

The typical symptoms of DM are excessive intake of water and food, urination, and weight loss, which are invisible in elderly populations. These symptoms can be important hints for diagnosis in young and middle-aged people, but in elderly people they are masked or manifested with other atypical symptoms due to a variety of factors such as reduced body functions due to natural aging, diseases, medications, and other factors.

It should be noted that polyuria due to DM is an increase in the volume of urine. Many diseases of the elderly, such as prostate enlargement in men and urinary tract infections in women, leads to an increase in the frequency of urination, which can be distinguished from polyuria due to DM. In addition to DM, excessive thirst and intake of water are also common in the elderly (El Osta et al., 2014; Islas-Granillo et al., 2017). Previous studies have shown that the majority of older adults experience hyposalivation or dry mouth, which may be associated with a decrease in the number of teeth in the mouth, aging, the female gender, and other diseases (hypertension, cardiovascular disease, neurologic disorders, and psychological disorders) (van der Putten et al., 2003; Flink et al., 2008; Abdullah, 2015; Ohara et al., 2016). Therefore, diseases with the same characteristics as diabetes tend to mask those symptoms of the elderly, which also shows the limitations of diabetes diagnosis relying on symptoms and physical examination screening.

In elderly groups, the syndromes of diabetes may manifest itself in other forms. Diminished pressure receptor-mediated regulation of thirst in that weakens physical function of Aging body, induces higher thirst osmolality set point (Koch and Fulop, 2017). The symptoms of excessive thirst and drinking could be replaced by fatigue and cognitive deficits (Hoen et al., 2021). Diabetes causes glycation of blood fibrinogen and decreased production of fibrinolytic enzymes, which directly affects fibrinolysis and leads to hypercoagulable state (Ajjan et al., 2013). Excessively coagulated blood increases the risk of stroke, acute coronary syndrome, or intermittent claudication in elderly. Thus, all of these atypical forms of morbidity may act as the initial symptoms of elderly diabetes (Mordarska and Godziejewska-Zawada, 2017).

### 2.2 High risk of hypoglycemia

Hypoglycemia is more common in the elderly diabetic populations. A cohort study based on 987 elderly diabetic patients showed that about one-third of the elderly patients have hypoglycemia, within 3.3% of cases were severe (Bordier et al., 2015). As well known, hypoglycemia in the elderly is closely related to anti-diabetes regimens like sulfonylureas and insulin (Ling et al., 2021).

Decreased autonomic function due to aging results in a reduced response intensity to hypoglycemic symptoms in elderly diabetic patients. n healthy individuals, aging also allows attenuation of blood glucose recovery and reductions of counter-regulatory responses. For example, emerging warning signals associated with hypoglycemia (such as sweating, shivering, or hunger) generated by stimulation of adrenergic system, may not be present in the elderly (Mordarska and Godziejewska-Zawada, 2017). In addition, negative feedback regulation to glucagon secretion often becomes inadequate and limited.

### 2.3 Increased incidence of complications and coexisting disorders

In the natural course of diabetes, the incidence of complications increases with the duration of diabetes, making elderly adults at high-risk for microvascular (retinopathy, nephropathy, neuropathy) and macrovascular (coronary heart disease, stroke, peripheral arterial disease) complications of DM (Gregg et al., 2002). It has been shown that the most common cardiovascular complications in elderly patients with diabetes are coronary heart disease, followed by lower extremity vascular disease, cerebrovascular disease, and heart failure (Sinclair et al., 2015; Bauduceau et al., 2018). Also, gradual loss of skeletal muscle mass, cognitive impairment, depression, urinary incontinence, falls, fractures, and other geriatric syndromes are often comorbid or concomitant. Elderly patients with T2DM have accelerated loss of lean leg mass, muscle strength and functional capacity compared to the normoglycemic group (Leenders et al., 2013). Plasma dipeptidyl peptidase-4 activity was shown to be independently associated with mild cognitive impairment in elderly T2DM (Zheng et al., 2016). Indian survey shows that nearly one-fifth of elderly diabetes patients are suffering from depression, urban living and financial support can prevent the occurrence of depression (Sodhi et al., 2023). Diabetes duration, neuropathy and albuminuria are risk factors for urinary incontinence in elderly women with diabetes and are particularly associated with severe incontinence (Vischer et al., 2009). Increased risk of hip fracture is seen primarily in patients treated with insulin, while T2DM patients treated with any glucose control medication are consistently at increased risk of non-skeletal fall injuries (Wallander et al., 2017).

Compared to those clinical features of young and middle-aged adults, the problems faced by elderly population, include the characteristics of invisible typical symptoms, high risk of hypoglycemia, and increased incidence of complications and coexisting disorders (macrovascular and microvascular lesions, sarcopenia, cognitive dysfunction, depression, urinary incontinence, falls, and fracture). For these reasons, screening, and prevention of progression from prediabetes to diabetes are particularly important. According to the guideline (LeRoith et al., 2019), fasting glucose and/or HbA1c screening is recommended every 2 years for elderly population without diabetes, lifestyle interventions for those with pre-diabetes, and 2-h oral glucose tolerance test for those with established diabetes. Elderly diabetes patients should also undergo fingertip glucose testing as part of their daily testing regimen and regular cognitive screening.

## 3 Pathogenesis of elderly diabetes

### 3.1 Gut microbiota disturbance

**FIGURE 1:**
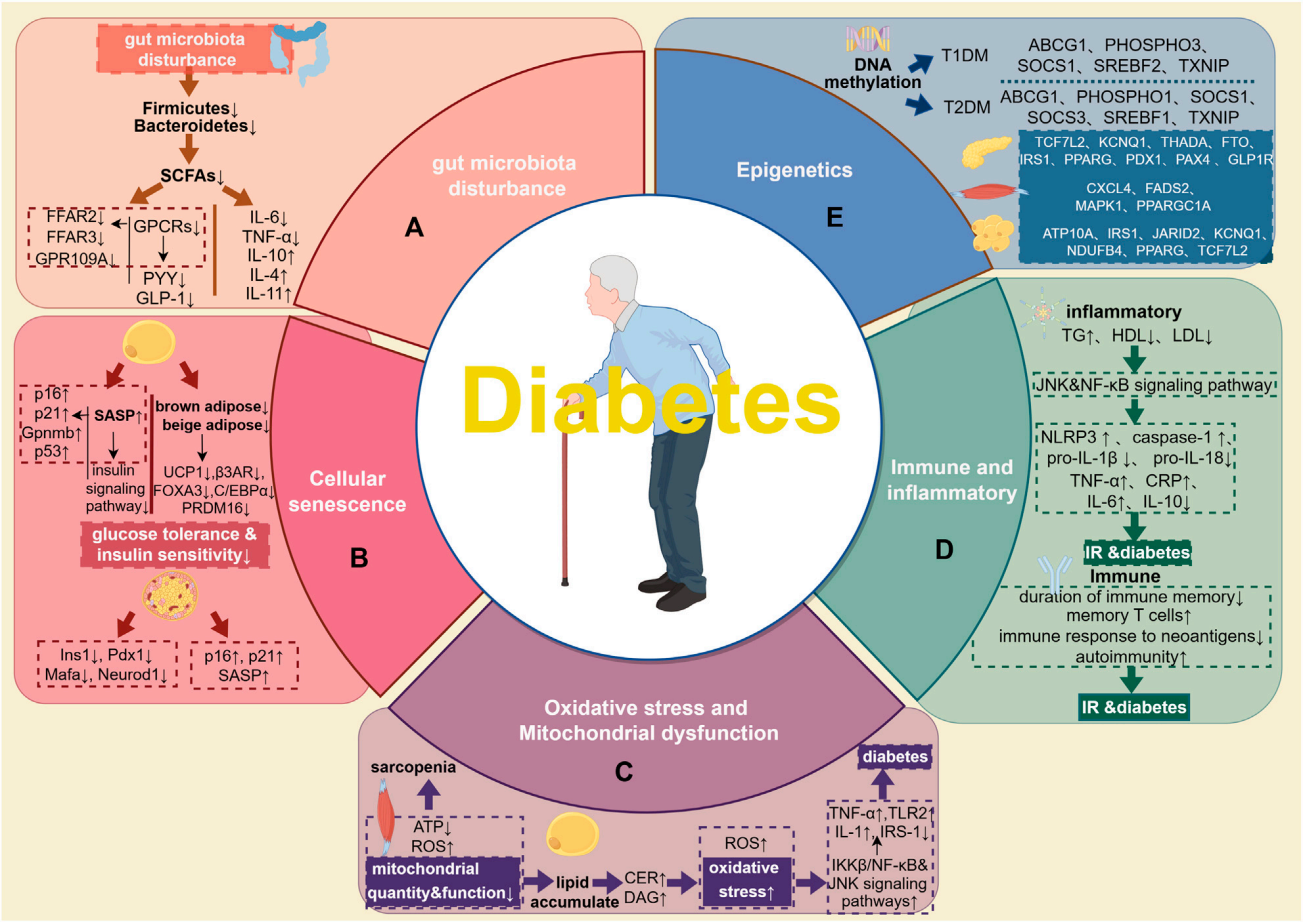
Pathogenesis of elderly diabetes. **(A)**, gut microbiota. The lack of *Firmicutes* and *Bacteroidetes* in the elderly may result in a decrease of SCAFs, and then put the intestines and pancreatic beta cells in a state of immune disorder and inflammation, leading to disorders of blood sugar homeostasis and metabolism. **(B)**, cellular senescence. Aging adipocytes disrupt the insulin signaling pathway by secreting SASP and regulating the expression of aging-related genes. At the same time, reduced volume of brown and beige adipose tissue and significantly reduced expression of genes related to thermogenesis and differentiation both jointly aggravate IR. In senescent islet cells, the expression of Ins1, Pdx1, Mafa, and Neurod1 decreases, and the expression of aging-related markers p16, p21, and SASP increases. **(C)**, oxidative stress, and mitochondrial dysfunction. Changes in skeletal muscle mitochondrial function, reduced ATP synthesis, and increased ROS production may be the causes of skeletal sarcopenia in the elderly with diabetes. Impaired mitochondrial oxidative function in skeletal muscle leads to excessive lipid deposition, increased CER and DAG, and further exacerbates oxidative stress, inflammation and diabetes. **(D)**, Immune and inflammatory. Elderly patients with diabetes are often accompanied by disorders of lipid metabolism, which activates related inflammatory pathways such as JNK and NF-κB, which in turn leads to the activation of pro-inflammatory factors such as NLRP3, caspase-1, TNF-a, CRP, IL-6 secretion and pro-IL-1β, pro-IL-18 cleavage increased. At the same time, the expression of anti-inflammatory factors such as IL-10 is reduced. In terms of immunity, the main manifestations are the shortened duration of immune memory, accumulation of memory T cells, the lack of immune response to neoantigens, and a higher tendency to autoimmunity, further induced the occurrence of IR and diabetes. **(E)**, Epigenetics. Epigenetic research on diabetes mostly focuses on DNA methylation. Methylation of ABCG1, P1, SHOSPHO3, SREBF1 and TXNIP is associated with a higher risk of T2DM; methylation of ABCG1, PHOSPHO3, SOCS1, SREBF2 and TXNIP is associated with T1DM. Different tissues have different methylation sites. In the pancreatic tissue of T2DM patients, the methylation sites are TCF7L2, KCNQ1, THADA, FTO, IRS1, PPARG, PDX1, PAX4 and GLP1R; in the skeletal muscle tissue, the methylation sites are CXCL4, FADS2, MAPK1, PPARGC1A and other sites; in adipose tissue, the methylation sites are ATP10A, IRS1, JARID2, KCNQ1, NDUFB4, PPARG and TCF7L2.

Pathogenesis of elderly diabetes (including Gut microbiota disturbance, Cellular senescence, Oxidative stress and mitochondrial dysfunction, Immune and inflammatory, and Epigenetics) has been summarized. Relevant details are indicated at Figure 1. A huge number of microorganisms such as bacteria, archaea, fungi, viruses, and phages habitats in gastrointestinal tract (Wang J. et al., 2023). The gut microbiota prototype forms prenatally, undergo rapid establishment during the neonatal and infancy stages, evolves with growth and becomes virtually stable in adulthood (Zhuang et al., 2019). Changes in the structure and quantity of gut microbiota, which serve as pathogenic mechanisms in chronic noncommunicable illnesses (Illiano et al., 2020), may have an impact on corresponding activities such as food digestion (Paone and Cani, 2020), energy metabolism (Li M. et al., 2022), immunological and genetic modulation (Wiertsema et al., 2021; Xu et al., 2023), as well as the integrity of the intestinal barrier (Adolph et al., 2019; Paone and Cani, 2020).

In 1907, Elie Metchnikoff postulated that one of the factors contributing to the decline in physical health was the gut microbiota and its metabolites (Cavaillon and Legout, 2016). In recent years, there has been a growing awareness of the correlation between gut microbiota and aging process. It had shown that gut microbiota was extremely different between the elderly and young population, manifested by a decrease in species diversity and probiotics, an increase in individual differences and harmful bacteria (Duncan and Flint, 2013; Mangiola et al., 2018). After comparing the gut microbiota between the elderly and young population in South Korea, Seung Yun Lee et al. found that *Firmicutes*, *Bacteroidetes*, *Cyanobacteria*, *Fusobacteria*, and *Proteobacteria* were more common in young people, while the abundance of *Negativicutes* was higher in the elderly (Lee et al., 2021). Furthermore, Tianyi Li et al. (Li et al., 2019) and Yongcheng Ni et al. (Ni et al., 2018) discovered that *Firmicutes* abundance was positively correlated with blood glucose level and sensitivity to insulin treatment in elderly T2DM patients, while the abundance of anaerobic bacteria (such as *Bacteroidetes*) was negatively correlated. *Firmicutes* are the major butyrate-producing bacteria in the human body, while *Bacteroidetes* are mainly acetate and propionate producers (Tsai et al., 2021). Previous research has revealed that short chain fatty acids, (SCFAs) including acetate, propionate (Chambers et al., 2015a; Wu et al., 2022), and butyrate (Gao et al., 2009; Chang et al., 2014; Mollica et al., 2017; Zhang et al., 2017; 2019; Li Z. et al., 2018; Stachowska et al., 2021) may target G protein-coupled receptors (GPCRs) such as FFAR2、FFAR3、GPR109A on the intestine, pancreatic islets, and immune cells, which may affect the metabolic function (Mayorga-Ramos et al., 2022). It has been demonstrated that SCFAs activate intestinal GPCRs to control the release of glucagon-like peptide 1 (GLP-1) and peptide YY (PYY) (Coppola et al., 2021), which in turn influences insulin secretion and the hypothalamic perception of energy intake. Furthermore, in pancreatic β-cell mitochondria, butyrate can suppress the expression of fission genes (DRP1, FIS1) and fusion genes (MFN1, MFN2, OPA1).

This can not only decrease the production of pro-inflammatory factors (such as IL-6 and TNF-α), but also increase the secretion of anti-inflammatory factors (such as IL-10, IL-4, and IL-11) (Mollica et al., 2017; Xiong et al., 2019; Noureldein et al., 2020; Prause et al., 2021). Hence, the lack of *Firmicutes* and *Bacteroidetes* in the elderly may result in a decrease of SCAFs, and then put the intestines and pancreatic beta cells in a state of immune disorder and inflammation, leading to disorders of blood sugar homeostasis and metabolism.

Besides SCFAs, Shin Yoshimoto et al. investigated metabolic products of gut microbiota in young and elderly groups found that choline, trimethylamine (TMA), N-8-acetylspermidine, 2-hydroxy-4-methylvaleric acid, and 5-methylcytosine were more abundant in elderly individuals (Yoshimoto et al., 2021). These metabolites have been proven to be high-risk factors for geriatric diseases such as metabolic syndrome (Chen et al., 2019; Zhou et al., 2021), cardiovascular disease (Nayak et al., 2020; Li et al., 2021), and tumors (Yin et al., 2022).

In elderly individuals, the administration of *Bifidobacterium* has been observed to regulate intestinal barrier function, exert anti-inflammatory and antioxidant effects, and result in an increased abundance of this organism in stool samples. These effects have been found to be associated with a reduction in fasting blood glucose levels and improvement in insulin resistance among patients diagnosed with T2DM (Schiffrin et al., 2007; Ouwehand et al., 2008). The utilization of *B subtilis natto DG101* in conjunction with suitable medications has demonstrated promising outcomes in the management of T2DM (Cardinali et al., 2020). Additionally, the potential cognitive benefits of probiotics can be attributed to their ability to reduce visceral fat and mitigate inflammation (Azuma et al., 2023).

### 3.2 Cellular senescence

Cellular senescence is a complicated yet ubiquitous process which is essential for biological embryonic development, tissue remodeling, and wound healing (Huang et al., 2022b). Cellular senescence is often distinguished by a reduction in the capacity for cell division, stoppage of the cell cycle, and the secretion of senescence-associated secretory phenotypes (SASP), which include cytokines, chemokines, growth factors, and proteases (Basisty et al., 2020). These chemicals can be delivered extracellularly via small vesicles, resulting in alterations to intercellular communication and the exertion of regulatory influences on adjacent cells, tissues, and even faraway tissues (Özcan et al., 2016; Terlecki-Zaniewicz et al., 2018). Nevertheless, the onset of age-related diseases is inextricably tied to the process of excessive and protracted senescence (Palmer et al., 2019; Yousefzadeh et al., 2021). Clinical studies revealed that increasing age is one of the risk factors for elderly diabetes, which is closely related to the aging of tissues and organs (Markle-Reid et al., 2018; Kumar et al., 2023).

Studies verified that the cellular senescence of diabetes target organs (such as the adipose tissue and pancreas) might accelerate the occurrence and progression of diabetes. Furthermore, obesity, lipid metabolism problems, and hyperglycemia also exacerbated this process (Narasimhan et al., 2021). Adipose tissue is crucial for storing energy, maintaining body temperature, safeguarding internal organs, and play a role in wound healing, immune as well as endocrine regulation. (Zwick et al., 2018). Nevertheless, the composition and functionality of adipose tissue undergo substantial alterations with aging or serious metabolic stress. These changes include the redistribution of body adipose, an increase in chronic aseptic inflammation, a decline in the function of adipose progenitor cells, heightened lipotoxicity of nearby tissues caused by ectopic fat deposition, diminished secretion and sensitivity of hormones derived from adipose tissue (Palmer and Kirkland, 2016). By secreting SASP and recruiting immune cells, ageing adipocytes may aggravate inflammatory responses and disrupt the insulin signaling pathway, which aggravate IR and raise the risk of T2DM and metabolic syndrome (Khosla et al., 2020). A comparison of adipose tissue between young and elderly people showed that the volume of brown and beige adipose tissue around shoulder blades, blood vessels, and kidneys was smaller in the elderly. Furthermore, the expression of adipose tissue thermogenesis and differentiation related genes (*UCP1, β3AR, FOXA3, C/EBPα* and *PRDM16*) significantly decreased (Ma et al., 2014; Wang et al., 2019; Ikeda and Yamada, 2020; Ou et al., 2022). This may cause the fibrosis of fat precursor cells and reduced the differentiation of beige cells in the elderly, causing more fat to be stored under the skin and around internal organs, inducing and enhancing the risk of metabolic diseases. Aside from functional alterations, adipocytes from elderly people and mice also show increased expression of aging-related genes such as *p16, p21, Gpnmb,* and *p53*. Activation and overexpression of *p53* can lead to DNA damage, downregulation of insulin signaling protein transcription, inflammatory response, and macrophage infiltration, which have been confirmed to be associated with reduced lipogenesis, IR, and the occurrence of diabetes (Vergoni et al., 2016; Lahalle et al., 2021). However, the mechanism of other aging molecules such as *p16* and *p21* induced IR and diabetes is still unclear.

Additionally, it has confirmed that the aging of pancreatic beta cells is also associated with elderly diabetes. Age-related alterations in pancreatic beta cell function include decreased insulin production, decreased beta cell mass, and delayed cell proliferation. These processes might be potential roads that lead to elderly diabetes (Aguayo-Mazzucato, 2020). Cristina Aguayo-Mazzucato et al. compared the pancreatic islets of young and elderly mice and recognized that senescent islet cells accumulated obviously in the elderly mice, companied the downregulation of *Ins1, Pdx1, Mafa, Neurod1*, while aging markers (*p16, p21*), SASP (*Ccl2, Il1a, Il6, Tnf*) expression increased (Aguayo-Mazzucato et al., 2019; Aguayo-Mazzucato, 2020). An analogous pattern can be observed in human beings, where the prevalence of senescent islet β cells will progressively escalate because of IR, obesity, and diabetes.

Intervention strategies in the cell senescence process may be able to ameliorate metabolic problems and the progression of diabetes in the elderly (Tchkonia et al., 2021). Suda et al. and Wang et al. have confirmed that reduced expression of senescent cells and senescence markers in adipose tissue can improve glucose tolerance and insulin sensitivity (Suda et al., 2021). Currently, commonly used anti-aging combination include Dasatinib plus Quercetin (D + Q). This solution can target senescent adipocyte progenitor cells, reducing adipose tissue inflammation, increasing adipose tissue and peripheral insulin sensitivity, as well as promoting adipocyte progenitor cell development, ultimately treating diabetes in the elderly (Hickson et al., 2019; Wang et al., 2022a).

### 3.3 Oxidative stress and mitochondrial dysfunction

Mitochondria are double-membraned organelles that play a pivotal role in ATP synthesis and energy metabolism. Fatty acids and glucose both contribute to energy metabolism via glycolysis and the tricarboxylic acid cycle. The majority of high-energy electrons are transmitted downstream after combining with the reduced coenzyme (NADH or FADH2) of the respiratory chain in the mitochondria. Thereafter, they combine with oxygen to produce water and ATP (Nolfi-Donegan et al., 2020; Prasun, 2020). However, some electrons are not transferred through the respiratory chain but react directly with oxygen to produce superoxide radicals and hydrogen peroxide (Addabbo et al., 2009). This in turn damages cell membranes, proteins, enzymes, and DNA, leading to cell death. When mitochondrial dysfunction occurs, excessive accumulation of ROS prevents superoxide dismutase and reducing glutathione peroxidase in mitochondria from exerting their antioxidant effects. This process is called oxidative stress (Nolfi-Donegan et al., 2020).

Mitochondrial function weakens with age, owing to lower antioxidant capacity, decreased ATP synthesis, mitochondrial DNA (mtDNA) damage, mitochondrial protein oxidation, decreased electron transport chain efficiency, and worse quality control during mitophagy (Chistiakov et al., 2014). Previous studies have confirmed that mitochondrial dysfunction and oxidative stress in target organs (e.g., skeletal muscle, adipose tissue) are related to elderly diabetes. Sarcopenia, in addition to typical microvascular and macrovascular disorders, has emerged as the third significant complication among older diabetes patients. It is a significant role in patients’ poor quality of life and impairment. Changes in skeletal muscle mitochondrial function, ATP reduced synthesis and increased ROS generation may be possible causes of sarcopenia in elderly diabetes (Izzo et al., 2021). Paul Coen et colleagues discovered that the quantity and function of mitochondria in skeletal muscle were significantly diminished in the elderly when compared to young persons, and that the number of mitochondria in skeletal muscle was positively connected with insulin sensitivity (Coen et al., 2013). Pelletier et al. discovered that the oxidative phosphorylation and oxidation capacities of elderly muscle were diminished, which inhibited oxidative breakdown of lipids in muscles then increased lipid accumulate (St-Jean-Pelletier et al., 2017). Lipid accumulation raises ceramide (CER) and diacylglycerol (DAG) levels, which lower the function of mitochondrial oxidative phosphorylation and disrupting the electron transport chain (Chaurasia and Summers, 2021). As a consequence, substantial volumes of ROS were produced. ROS production activates downstream targets such as the IKKβ/NF-κB and JNK signaling pathways, increases inflammatory factor secretion (TNF-α, TLR2, IL-1), decreases IRS-1 activity, and affects insulin signaling (Michot et al., 2013). Increased levels of inflammation diminish IRS-1 expression and disrupt insulin signaling, resulting in elderly diabetes. Through comparing adipose tissue from young and elderly people, Laura Pelletier et al. found that mitochondrial dysfunction, oxidative stress, and adipocyte dysfunction were more obvious in elderly adipose tissue which accompanied by impaired lipogenesis and insulin sensitivity (L et al., 2021).

### 3.4 Immune and inflammatory

There are two types of immune systems: innate immunity and adaptive immunity. The complement system and various types of white blood cells (natural killer cells, mast cells, eosinophils, basophils, phagocytic cells, macrophages, neutrophils, and dendritic cells) comprise the innate immune system, which can produce cytokines and recruit immune cells to the site of infection or inflammation, then activate the adaptive immune process via antigen presentation (Daryabor et al., 2020). Adaptive immune cells include B cells and T cells, which mainly participate in humoral immunity and cellular immunity processes (SantaCruz-Calvo et al., 2022). A great number of research over the last 2 decades have demonstrated that immunometabolism is a key mechanism for controlling adaptive and innate immunity (Makowski et al., 2020). That is, metabolic processes can influence immune cell activity, and immunological and inflammatory responses may be the root causes of metabolic diseases.

However, as age increases, the immune system of the elderly will show a senescence state, which is manifested by a shortened duration of immune memory, accumulation of memory T cells, a lack of immune response to neoantigens, a higher tendency to autoimmunity, and a persistent low-grade inflammatory state throughout the body (Franceschi et al., 2018; Bulut et al., 2020; Moskalev et al., 2020). Inflammation can be caused by a variety of factors, including heightened levels of proinflammatory cytokines in the blood flow, disrupted lipid metabolism, alterations in the composition of gut microbiota, compromised immune system functioning, and the presence of meta-inflammation (Singh and Newman, 2011).

Previous studies have confirmed that the level of cellular inflammatory factors such as tumor necrosis factor-α (TNF-α), interleukin-6 (IL-6) and C-reactive protein (CRP) was increased in elderly patients with diabetes, and anti-inflammatory factors such as IL-10 were reduced (Gorska-Ciebiada et al., 2015; Saukkonen et al., 2018; Yang et al., 2018; Leite et al., 2021). These inflammatory factors have been confirmed to have a negative effect on insulin production, secretion and insulin signaling pathway (Shoelson et al., 2006; Akbari and Hassan-Zadeh, 2018), which may induce programmed cell death of pancreatic β cells (Maedler et al., 2009), and aggravate the risk of IR and diabetes. (Wang et al., 2013).

Furthermore, elderly patients with diabetes are often accompanied by abnormalities in lipid metabolism, mainly manifested by high triglycerides, low high-density lipoprotein cholesterol, and elevated small-density low-density lipoprotein cholesterol. (Gyawali et al., 2018). These pro-inflammatory lipids result in the activation of inflammatory signaling pathways, including JNK and NF-κB (Osborn and Olefsky, 2012; Tall and Yvan-Charvet, 2015). Activation of inflammatory signaling pathways lead to increased metabolic inflammatory stress (Hotamisligil, 2017) and promote the activation of NLRP3 (Masters et al., 2010; Wen et al., 2011; Wen et al., 2013) and caspase-1 as well as the cleavage of pro-IL-1β and pro-IL-18 (Wen et al., 2012), thereby aggravating systemic chronic inflammation and inducing the occurrence of IR (Masters et al., 2010).

Anti-inflammation and regulating immune cell function are ways to alleviate insulin resistance and pancreatic β-cell dysfunction. Commonly used drugs include metformin, GLP-1 receptor agonists (GLP-1 RAs) and dipeptidyl peptidase 4 (DPP4) inhibitors (Jo and Fang, 2021). In addition, IL-1R antagonists can reduce islet inflammation caused by hyperglycemia, thereby improving islet β-cell dysfunction, alleviating insulin resistance and blood sugar homeostasis. However, the clinical efficacy of the above-mentioned drugs for elderly diabetes patients remains to be seen due to a lack of clinical research.

### 3.5 Epigenetics

Epigenetics is the study of heritable changes in the genetic information of gene-related characteristics that do not involve in modifying the DNA sequence, such as DNA methylation, histone modifications, and non-coding RNA (Allis and Jenuwein, 2016; Ling and Rönn, 2019). Furthermore, several enzymes are involved in post-transcriptional protein modification, such as acetylation, methylation, phosphorylation, and ubiquitination. However, most clinical studies have shown that there is a close relationship between chromatin formation, histone modifications, DNA methylation and gene activity, which have differential epigenetic changes expressed in different target tissues. So, it is difficult to determine which epigenetic phenomenon appears first.

The direct effects of aging on metabolic regulation exacerbate the underlying pathophysiological processes in elderly patients with diabetes. Aging effects interact with diabetes to accelerate the progression of many common diabetes complications (LeRoith et al., 2019). This is often associated with varying degrees of underlying IR, excess obesity, beta cell dysfunction, and sarcopenia, and increase the complexity of diabetes management in this age group (Bellary et al., 2021). Most of the current epigenetics research in diabetes are focused on DNA methylation. Previous studies have shown that the methylation patterns of diabetic patients are significantly different from those of healthy population, and there are also obvious differences in the methylation sites of different target tissues in patients (Singh et al., 2020). John Chambers et al. followed 25,372 Indian Asians and Europeans and found that methylation of ABCG1, PHOSPHO1, SOCS3, SREBF1 and TXNIP in the blood was associated with a higher risk of future T2DM; in type 1 diabetes Methylation of ABCG1, PHOSPHO3, SOCS1, SREBF2 and TXNIP sites was found (Chambers et al., 2015b). But when it comes to specific tissues, their methylation sites are different. When comparing pancreatic tissue from healthy people and T2D patients, multiple studies have found that TCF7L2, KCNQ1, THADA, FTO, IRS1, PPARG, PDX1, PAX4, and GLP1R sites are methylated, which may be related to the methylation of pancreatic islet β after transcriptional inactivation. Decreased cell secretory function and activation of pro-inflammatory pathways (Dayeh et al., 2016; Suárez et al., 2023). In skeletal muscle, CXCL4, FADS2, MAPK1, PPARGC1A and other sites; in adipose tissue, it shows methylation of ATP10A, IRS1, JARID2, KCNQ1, NDUFB4, PPARG, and TCF7L2.

## 4 Treatment principles of elderly diabetes

With the decline of pancreatic beta cell function and the increase of IR, the prevalence of diabetes is gradually increasing in the elderly (Motta et al., 2008a; Tekin and Zimmerman, 2020). Common comorbidities in elderly patients with diabetes include chronic kidney disease, cognitive impairment, chronic airway disease, and infection (Fagot-Campagna et al., 2005; Lin et al., 2016). Multiple medications may have adverse effects (Noale et al., 2016), so appropriate management of comorbidities should be included in the guidelines for elderly diabetes patients (Caughey et al., 2010). The challenge of managing elderly diabetes patients is that it is highly heterogeneous (Bennett, 2015), which requires personal assessment of treatment and care options (Munshi et al., 2020), as well as comprehensive education of patients (Tekin and Zimmerman, 2020).

### 4.1 Western medicine treatment principles and clinical intervention

The target level of blood glucose and the use of anti-diabetes medications should be comprehensively evaluated according to clinical status, risk of hypoglycemia, and complications of diabetes (Scheen et al., 2014). Failure of oral glycemic control therapy is often observed in elderly patients, so insulin is often chosen as the preferred medication (Rajpal et al., 2021). However, the dangers associated with hypoglycemia cannot be ignored. Regimens using DPP-4 inhibitors alone or in combination with basal insulin have been shown to be safe and effective and may be an alternative to basal injection regimens in elderly patients (Umpierrez and Pasquel, 2017). Reducing the frequency and severity of hypoglycemia is the key to achieving better compliance in elderly patients with diabetes (Motta et al., 2008b). In addition, the participation of family members is the basis for the good treatment effect of elderly diabetic patients (Baig et al., 2015). Besides, aiming to maintain or improve general health, the management goals of elderly patients with diabetes also include the assessment and treatment of atherosclerosis and microvascular disease (Adu-Sarkodie, 2017).

Specific clinical guidelines for T2DM in elderly have been published in Europe and the United States, but they do not specifically address advanced chronic kidney disease in elderly people with diabetes. Elderly patients with diabetes are different from younger patients, mainly due to their frailty and shorter life expectancy, requiring different treatment strategies to be tailored (Abaterusso et al., 2008). Studies have found that in the treatment of elderly diabetic nephropathy, excessive reduction of blood pressure to the current target is unsafe (Williams, 2013). The selection of some specific medications could improve the cure rate, reduce blood glucose, and improve kidney function for diabetic nephropathy in elderly (Cao and Chen, 2021).

Diabetic peripheral neuropathy (DPN) is the main form of neuropathy and a leading cause of disability. Small fiber neuropathy (SFN) can develop in older people with pre-diabetes, prior to large fiber damage (Akbar et al., 2023). The symptoms of DPN consist primarily of spontaneous, intractable pain that is diffuse and persistent and can last for weeks to months. Clinical treatment focuses on alleviating clinical symptoms, improving blood glucose control and cardiovascular risk factors (Yang et al., 2022). Pain in the elderly need arouse attention from clinicians and patients (Marchesi et al., 2024). In pain management, anticonvulsants such as pregabalin and gabapentin are the first-line treatment of choice, followed by amitriptyline, duloxetine, and venlafaxine (Cernea and Raz, 2021). Opioids and related medications are recommended for short-term use during acute exacerbations of pain (Kozma et al., 2012; Feldman et al., 2019; Marchesi et al., 2024). These interventions play an important role in diminishing DPN’s symptoms and complications. For patients who do not respond to monotherapy, combination therapy may be beneficial (Rafiullah and Siddiqui, 2022). Basic interventions also include nutritional recommendations (mecobalamin, etc.) and functional exercise (Didangelos et al., 2020; 2021; Seyedizadeh et al., 2020; Shen et al., 2023b; Marchesi et al., 2024). However, diet and exercise are often neglected in the treatment of elderly patients with diabetes. Nutritional recommendations and exercise resistance training based on elderly subjects to increase muscle mass have good value in reducing diabetes parameters (Constans and Lecomte, 2007).

### 4.2 TCM treatment principles

The comprehensive and holistic management with TCM for diabetes patients is gradually being favored by modern healthcare systems (Xiao and Luo, 2018). The combination of TCM characteristics and western medicine to prevent and treat diabetes becomes a new attempt (Wang et al., 2020b). More and more studies have emphasized that the bioactive components of TCM participate in those mechanism mentioned above (Nie et al., 2019; Ai et al., 2020; Tang et al., 2021; Huang et al., 2022c). TCM emphasizes Yin and Yang balance and a holistic approach. Chinese herbal medicine bidirectional regulation of body metabolism, to maintain the balance of the body’s internal environment. TCM individual treatment focuses on syndrome differentiation, multi-level and multi-target treatment, and patients with diabetes can significantly relieve symptoms under the treatment of TCM theory (Tong et al., 2012).

Clinical TCM classifies diabetes as “Xiaoke” and “Pidan”. Some scholars put forward the idea of “state-target” differentiation and treatment (Zhang et al., 2021a), and believe that the stage identification and treatment of elderly diabetes are different from the general diabetes population. According to the different stages of clinical manifestations and pathological changes, syndrome differentiation and treatment can alleviate the symptoms and physique of patients (Wang et al., 2020b). The pathological characteristics of elderly patients with diabetes are gradually becoming weak, accompanied by insufficient digestive ability. Due to long-term nutritional metabolism deficit, often just at the beginning of the disease will see the phenomenon of physical weakness, so elderly diabetes is easy to enter the stage of deficiency, damage, often combined with a variety of complications. TCM believes that most elderly patients are sick for too long and lead to physical weakness, with the gradual loss of Yang physical characteristics, so strong Yang is often the treatment principle of elderly diseases.

TCM believes that most elderly patients have a long course of disease, which leads to physical weakness and gradual imbalance of Qi, Blood, Yin, and Yang. Therefore, the use of TCM intervention strategies, focusing on improving immunity, improving circulation, and reducing inflammation may becoming the key to the treatment of elderly diabetes.

## 5 TCM treatments of elderly diabetes

Over the past few years, numerous investigations have been carried out to find evidence-based anti-diabetes TCM formulas but few for elderly diabetes. We conducted a literature review on PubMed and CNKI updated until November 2023, for eligible studies on the Traditional Medicine accepted for use in clinical settings by the elderly population with diabetes or diabetic complications. And we searched keywords and Medical Subject Headings (MeSH) terms pertinent to the intervention of interest, such as “elderly diabetes”, and “Traditional Medicine”. All involved TCM interventions for elderly diabetes were summarized (Table 1).

**TABLE 1:** Clinical trials of TCM in complications with elderly diabetes.

Disease	Reported TCM intervention in elderly diabetes	Ref. #
Breast cancer	Di Huang Wan series	Wu et al. (2018)
Hyperglycemia	health management, TCM dietary scheme, TCM syndrome differentiation and treatment, Sini decoction, TCM tea	Xu, 2019; Zhuang (2019), Wang, (2020); Wu et al. (2021), Li Y. et al. (2022), Lin et al. (2023)
IR	Goshajinkigan, Jinlida, TCM syndrome differentiation and treatment	Uno et al. (2005), Tian et al. (2018), Tsai et al. (2020)
CHD and Atherosclerosis	Acupuncture, TCM syndrome differentiation and treatment	Zhang et al. (2008), Fang et al. (2015)
Diabetic foot	ARCC (Angelica, Angelica, Calcined Gypsum and Caleramide), NF3 (Astragali Radix and Radix Rehmanniae)	Ko et al. (2014), Zhong et al. (2022)
DKD	Liu-Wei-Di-Huang-Wan, Qi-Kui granules, TCM syndrome differentiation and treatment	Gong, 2013; Hsu et al. (2014), Wang L. et al. (2023)
DPN	Acupuncture	Meyer-Hamme et al. (2021)
Stroke	TCM syndrome differentiation and treatment, Bu Yang Huan Wu Tang, Tongluo Xifeng formula	Ma and Xia (2017), Tsai et al. (2017), Chiao et al. (2018), Weng et al. (2021)
DR	Mingmu Dihuang decoction, Zhenwu decoction	Zhong, 2018; Li (2022)
Hyperhidrosis	Yupingfeng power	Chang et al. (2021)
Constipation	Modified Sini power, modified Jichuan decoction	Feng and Tong (2009), Li T. et al. (2018)

Abbreviations: Diabetic peripheral neuropathy (DPN); Diabetic kidney disease (DKD); Insulin resistance (IR); Traditional Chinese Medicine (TCM); Diabetic retinopathy (DR).

Though there were insufficient direct clinical proofs on TCM for diabetes and related complications in elderly patients, it is beyond question that TCM addresses the health of the population. Moreover, TCM have provide patient-centered treatment strategies for blood glucose problem that can partly replace western medicine. A meta-analysis reported that TCM could significantly improve glucose control and clinical indices in patients with diabetes and effectively delay the progression of diabetes (Tian et al., 2019). As early as 2015, the first RCT on TCM formula for diabetes and mechanism exploration with gut microbiota was conducted, providing powerful clinical proofs on this issue (Xu et al., 2015). A traditional Chinese herbal formula, Gegen Qinlian Decoction, can exert similar diabetes-control effects with metformin in a dose-dependent manner (Tong et al., 2011; Xu et al., 2020; Tian et al., 2021).

Also, studies reported in Chinese concentrated on senile disease (such as DKD) and other common symptoms or signs in elderly diabetes like constipation and diabetic gastroparesis. It is obvious that the danger and particularity of elderly diabetes have attracted more attention in recent years, and we’ve also just seen the size of these settlements balloon. Various TCM interventions could solve a majority of problems for elderly diabetes. And different complications suit for different TCM interventions. For example, acupuncture plays a crucial role on diabetic peripheral neuropathy while TCM exercise therapy (such as Taichi) helps weight loss and muscle function recovery (Jiang et al., 2020; Huang. et al., 2022a; Shen et al., 2023b; Li et al., 2023).

Clinical trials on TCM intervention in elderly diabetes was also retrieved from WHO International Clinical Trials Registry Platform (https://www.who.int/clinical-trials-registry-platform), ClinicalTrials.gov. (https://clinicaltrials.gov/). and Chinese Clinical Trial Registry (https://www.chictr.org.cn/). We noticed that there was an increasing trend in trials on TCM intervention in elderly diabetes (Table 2). Such TCM treatment of elderly diabetes research is currently in progress.

**TABLE 2:** Registered clinical trials on the elderly diabetes.

Main ID	Country	TCM intervention	Disease	Expected endpoints	Status
IRCT20201128049510N1	Iran	Mulberry leaf extract	Elderly diabetes	cardiovascular inflammatory markers	Recruiting
ChiCTR2300075132	China	Bushen Tongmai Recipe	Elderly diabetes complicated with ASCVD	Blood lipids, Crouse plaque points, inflammation factor	Pending
RBR-75qb7pp	Brazil	curcumin supplementation	elderly women	glycemic levels, lipid profile and body weight	Recruitment completed
ITMCTR2000003276	China	Sancai powder	Elderly diabetes	blood glucose level, glycemic variability	Pending
ChiCTR2000039049	China	Yi-Jin-Jing	Middle-aged and elderly obese people with pre-Diabetes	Bone mineral density, FGF23 and other markers of bone metabolism	Recruiting
ChiCTR1800020069	China	Qigong and Tai Chi	Middle-aged and elderly diabetes	glycemic levels, C-peptide	Completed
IRCT2015080822466N2	Iran	Herbal combination	pre-diabetic elderly	glycemic levels	Completed
RBR-2sgtn2	Brazil	Green Tea	elderly Metabolic Syndrome	abdominal circumference	Not yet recruiting

**FIGURE 2:**
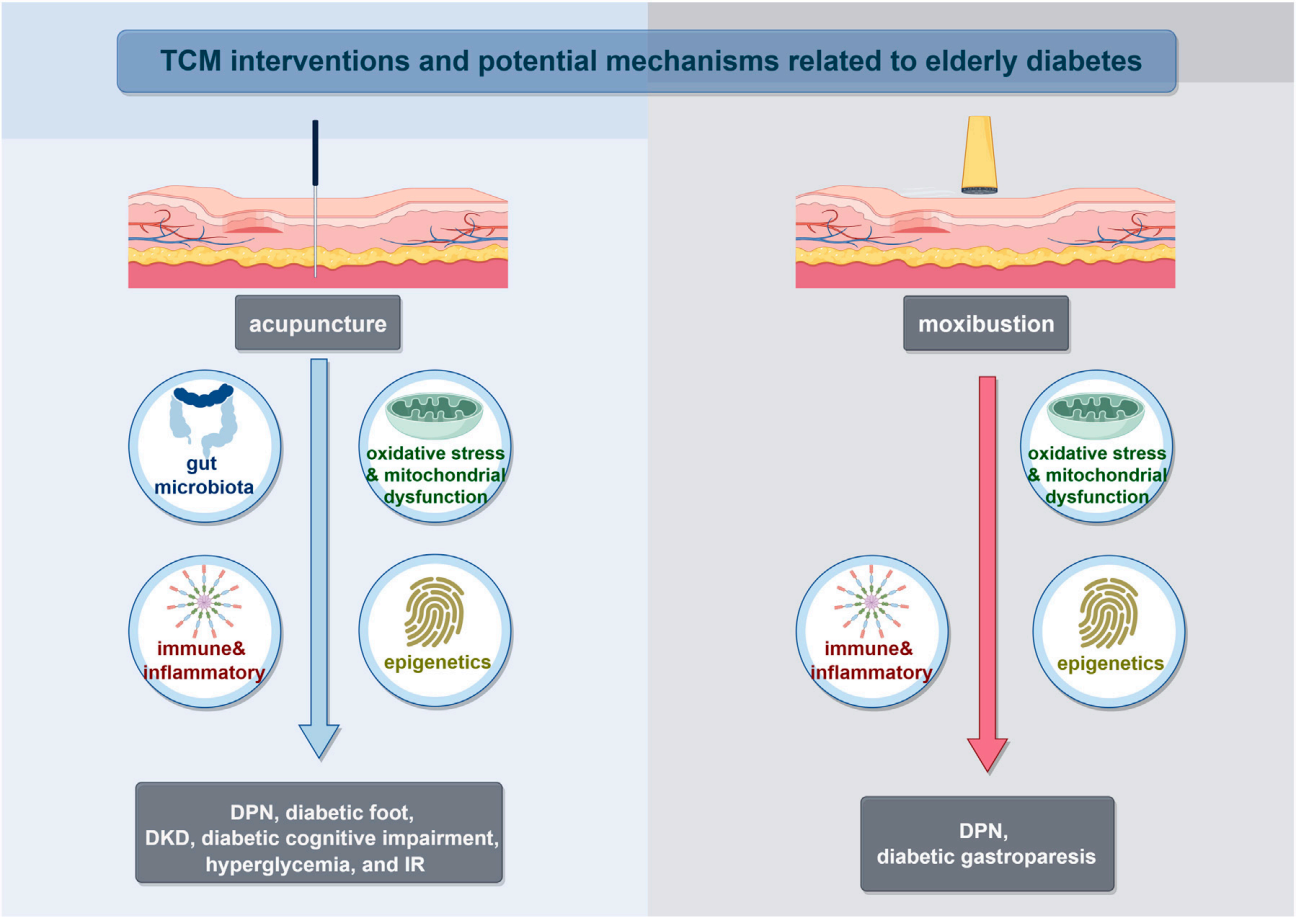
TCM interventions and potential mechanisms related to elderly diabetes. Abbreviations: Diabetic peripheral neuropathy (DPN); Diabetic kidney disease (DKD); Insulin resistance (IR).

Furthermore, we have reviewed the common mechanism of TCM reported for aging and diabetes, or direct mechanism for elderly diabetes mentioned above. So far, those mechanism could be well implemented by distinctive TCM non-pharmacological approaches (Figure 2) and herbal treatment (Table 3). TCM had the effect of modulating gut microbiota and improving glucose metabolisms in T2DM patients and pre-clinical experiments (Zheng et al., 2021b). Numerous studies reviewed the efficacy and mechanisms of Chinese herbal medicine on DKD, DR, and DPN, which have been reported to be critical for diabetes in the elderly (Lu et al., 2019; Ai et al., 2020; Chen et al., 2022b; Liu et al., 2022). Whlie TCM could give full play to the advantages of multiple targets and intervene in many important mechanisms of elderly diabetes. Take Gegen Qinlian Decoction (GQD) as an example, its prescirption may ameliorate T2DM with hyperlipidemia via enriching beneficial gut microbiota (such as *Blautia* and *Faecalibacterium spp*) and decline harmful gut microbiota which closely related to the occurrence and development of T2DM (Tong et al., 2018). Also, the exosomal miRNA expression profile and signaling pathways related to T2DM was changed obviously following GQD treatment to provide a potential strategy for elderly diabetes. For elderly DKD, Dihong formula, Qi-Kui granules, and treatment based on syndrome differentiation were reported to exert the effect of oxidative stress inhibition and kidney protection.

**TABLE 3:** Representative examples of major anti-diabetic effects of TCM ingredients, herbs and formulations, as well as potential mechanisms related to elderly diabetes.

Bioactive ingredients	Representive herbs	Related formulations	Disease	Beneficial effects	Potential mechanism	Ref. #
Berberine	Huanglian	Gegen Qinlian decoction, Fufang Zhenzhu Tiaozhi capsule, Qijian mixture, Huanglian jiedu decoction	DPN, coronary atherosclerosis, DKD, DCD	modulating inflammation, alleviating apoptosis, and inhibiting EndMT of coronary artery, regulate mitochondrial energy homeostasis	①②③④⑤	Gao et al. (2018), Dong et al. (2019), Meng et al. (2021), Wang et al. (2022b)
Quercetin	-	Not available	DPN	reduces inflammation	①②③④⑤	B et al., 2021; Dhanya (2022), Roshanravan et al. (2023)
Resveratrol	-	Not available	DKD, CHD, obesity, and IR	improves intestinal barrier function and ameliorates intestinal permeability and inflammation	①②③④⑤	Cai et al. (2020), Huang et al. (2020)
Ginsenoside	Panax ginseng, Panax notoginseng	Baihu renshen decoction, Dan-qi prescription, Huanglian Maidong Decoction	Pre-diabetes, hyperglucose, IR and obesity	anti-diabetic, anti-hyperlipidemic, anti-inflammatory, and hepatoprotective	①②③④⑤	Xie et al. (2015), Fan et al. (2019), Zhou et al. (2019), He et al. (2021), Naseri et al. (2022)
ML polysaccharides	mulberry (Morus alba L.) leaves	Sansang Buxu decoction	hyper glucose, and IR	prevent glucolipid metabolism disorders, alleviating liver and kidney damage	①②③④⑤	Chen et al. (2022a), Tang et al. (2023)
Astragaloside	Astragalus membranaceus (Fisch.) Bunge	Huangqi Guizhi Wuwu decoction, Huangqi decoction, Qidan junzhi decoction	DPN, DKD, DR	antioxidant, anti-inflammatory, anti-apoptotic properties, and the roles in enhancement of immunity, attenuation of the migration and invasion of cancer cells and improvement of chemosensitivity	①②③④⑤	Shen et al. (2023a)
Tanshinone	Salvia miltiorrhiza	Danshenyin, Danggui Buxue Decoction, Compound Danshen Dripping Pills	DKD, DR	anti-oxidative stress, anti-inflammatory, anti-EMT effects	③④	Wang et al. (2020a), Zhang et al. (2021b), Guo et al. (2021), Sun et al. (2022)
Wogonin	Scutellaria baicalensis Georgi	Tourexiaozhen formula	DKD	anti-inflammatory, anti-apoptotic, anti-oxidative, and cell cycle regulatory effects	②③④⑤	Wang et al. (2018)

Note: ① Gut microbiota, ② Cellular senescence, ③ Oxidative stress and Mitochondrial dysfunction, ④ Immune and inflammatory, ⑤ Epigenetics.

Abbreviations: Diabetic peripheral neuropathy (DPN); Diabetic kidney disease (DKD); Insulin resistance (IR); Epithelial-mesenchymal transition (EMT); Diabetic cognitive dysfunction (DC); Coronary heart disease (CHD).

There were also various natural products (including oleuropein, cyclocarya paliurus polysaccharides, hyperoside, and so on) that do not used in traditional clinical practice, while existed proofs have reported excellent potentials for elderly diabetes considering consistent curative mechanism (Yao et al., 2020; Zheng et al., 2021a; Liu et al., 2021). Quercetin, an important natural flavonoid, not only regulate gut microbiota disorders in diabetes animal models, but also inhibiting oxidative stress and inflammatory responses to restore mitochondrial dysfunction (Yi et al., 2021; Cui et al., 2022). Moreover, there are numerous natural products from TCM used for various diabetes related complications, including DR, DPN and so on. Furthermore, we categorized and analyzed their main bioactive ingredients (Table 3). This review partly covers the key works on the effects and underlying mechanisms of TCM, herbal ingredients and synergistic effects of constituent compatibility in treating elderly diabetes, providing additional ideas to address this threat.

## 6 Discussion and prospects

Diabetes is a systemic chronic metabolic disease caused by a combination of genetic, nutritional, environmental, and other factors. With the improvement of living circumstances and the aging of population, diabetes is affecting an increasing number of senior individuals. As a result, early and thorough interventions have substantial therapeutic implications for delaying diabetes development, preserving target organs, preventing complications, and increasing patients' quality of life. Even though a range of medicines is being utilized to treat diabetes, current diabetes management in the elderly is still inadequate.

Diabetic patients in China frequently undergo TCM treatment in addition to conventional care, and the results are frequently superior to conventional treatment alone. Multiple studies have demonstrated that TCM can relieve clinical symptoms and postpone the development of diabetes by regulating exosome secretion and epigenetic expression, as well as enhancing gut microbiota and eliminating oxidative stress. However, due to the unique diagnostic methods of TCM and the complexity of TCM components, as well as the fact that the pathogenesis of diabetes in the elderly differs from that of other age groups, there is still a lack of large-scale, multi-center, randomized, and controlled clinical trials of TCM in the treatment of elderly diabetes. Furthermore, clinical studies have yet to validate several results, although clinical trials have proven improvements in clinical symptoms, the fundamental mechanisms remain unknown. To overcome these limitations, we should conduct additional analyses and searches for key compounds and targets of TCM in the treatment of elderly diabetes. Also, there is an urgent need to clarify the drug dose-response relationship and ensure the reliability of the results through experimental verification in the future. Besides, a high-quality TCM clinical research protocol should be established to facilitate the conduction of large-scale, multi-center, controlled trials, so that to provide stronger evidence for TCM treatment of diabetes in the elderly.
