# Supplementary material for: Characteristics of elderly diabetes patients: focus on clinical manifestation, pathogenic mechanism, and the role of traditional Chinese medicine
Source: Front Pharmacol. 2024 Jan 11;14:1339744. doi: 10.3389/fphar.2023.1339744 (PMC10808572; doi:10.3389/fphar.2023.1339744)
Supplement: Supplementary file 1 [file Table1.DOCX]

Table. S1. Mentioned TCM formulations for elderly diabetes patients in the text and their composition.

| Name | Composition |
| --- | --- |
| Liu-Wei-Di-Huang-Wan | processed radix of Rehmannia glutinosa Libosch (Shu Di Huang), rhizome of Dioscorea opposita Thunb. (Shan Yao), fructus of Corni officinalis, Sieb. et Zucc. (Shan Zhu Yu), sclerotium of Poriae cocos (Schw.) Wolf (Fu Ling), rhizoma of Alisma orientalis (Sam) Juzep. (Ze Xie), cortex radices of Moutan suffruticosa Andr. (Dan Pi) |
| Sini decoction | *Aconitum carmichaelii* Debeaux (Fu Zi), *Zingiber officinale* Roscoe (Gan Jiang), and *Glycyrrhiza uralensis* Fisch (Gan Cao) |
| Jinlida | Radix Ginseng (Panax ginseng C.A.Mey.) (Ren Shen), Rhizoma Polygonati (Polygonatum kingianum Coll. et Hemal.) (Huang Jing), Rhizoma Atractylodis (Atractylodes lancea (Thunb.) DC.) (Cang Zhu), Radix Sophorae Flavescentis (Sophora flavescens Ait.) (Ku Shen), Radix Ophiopogonis (Ophiopogon japonicus (Thunb.) Ker-Gawl.) (Mai Dong), Radix Rehmanniae (Rehmannia glutinosa Libosch.) (Di Huang), Radix Polygoni Multiflori (Polygonum multiflorum Thunb.) (He Shou Wu), Fructus Corni (Cornus officinalis Sieb. et Zucc.) (Shan Zhu Yu), Poria (Poria cocos (Schw.) Wolf.) (Fu Ling), Herba Eupatorii (Eupatorium fortunei Turcz.) (Pei Lan), Rhizoma Coptidis (Coptis chinensis Franch.) (Huang Lian), Rhizoma Anemarrhenae (Anemarrhena asphodeloides Bge.) (Zhi Mu), Herba Epimedii (Epimedium brevicornum Maxim.) (Yin Yang Huo), Radix Salviae Miltiorrhizae (Salvia miltiorrhiza Bge.) (Dan Shen), Radix Puerariae (Pueraria lobata (Willd.) Ohwi.) (Ge Gen), Semen Litchi (Litchi chinensis Sonn.) (Li Zhi He) and Cortex Lycii (Lycium chinense Mill.) (Di Gu Pi) |
| Bu Yang Huan Wu Tang | Astragalus membranaceus (Huang Qi), Angelica sinensis (Dang Gui), Paeonia lactiflora (Shao Yao), Ligusticum chuanxiong (Chuan Xiong), Carthamus tinctorius (Hong Hua), Amygdalus persica (Tao Ren) and Pheretima aspergillum (Di Long) |
| Tongluo Xifeng formula | Gastrodia elata Blume (G. elata, tianma) (Tian Ma), Ligusticum chuanxiong (Chuan Xiong), Carthamus tinctorius (Hong Hua), Radix Ophiopogonis (Ophiopogon japonicus (Thunb.) Ker-Gawl.) (Mai Dong), Red ginseng polysaccharide (Hong Shen), Radix Puerariae (Pueraria lobata (Willd.) Ohwi.) (Ge Gen) and *Glycyrrhiza uralensis* Fisch (Gan Cao) |
| Zhenwu decoction | Poria Poria cocos (Schw.) Wolf (Fu Ling), Paeoniae radix alba (Paeonia lactiflora Pall.) (Bai Shao),Zingiberis rhizoma recens (Zingiber officinale Rosc.) (Sheng Jiang), Aconiti lateralis radix praeparata (Aconitum carmichaelii Debx.) (Fu Zi), Atractylodis macrocephalae rhizome (Atractylodes macrocephala koidz) (Bai Zhu) |
| Yupingfeng power | Astragalus membranaceus (Huang Qi), Atractylodis macrocephalae rhizome (Atractylodes macrocephala koidz) (Bai Zhu), Radix Saposhnikoviae (R. Saposhnikoviae) (Fang Feng） |
| Jichuan decoction | Angelica sinensis (Dang Gui), C. deserticola (Rou Cong Rong), Achyranthes bidentata (A. bidentata) (Niu Xi), Fructus aurantii (F. aurantii) (Zhi Shi), Alisma orientalis (Chan Tui) and Cimicifuga heracleifuga (Sheng Ma) |
| Gegen Qinlian decoction | Rhizoma Coptidis (Coptis chinensis Franch.) (Huang Lian), Radix Puerariae (Pueraria lobata (Willd.) Ohwi.) (Ge Gen), Scutellaria baicalensis Georgi. (Lamiaceae) (Huang Qin), *Glycyrrhiza uralensis* Fisch (Gan Cao) |
| Qijian mixture | Astragalus membranaceus (Huang Qi), Ramulus euonymi (Gui Jian Yu), Rhizoma Coptidis (Coptis chinensis Franch.) (Huang Lian), Radix Puerariae (Pueraria lobata (Willd.) Ohwi.) (Ge Gen) |
| Huanglian jiedu decoction | Coptidis Rhizoma (Huang Lian), Scutellariae Radix (Huang Qin), Phellodendri Chinensis Cortex (Huang Bai), Gardniae Fructus (Zhi Zi) |
| Dan-qi prescription | Salviae miltiorrhizae Radix et Rhizoma (Dan Shen) and Notoginseng Radix et rhizome (San Qi) |
| Huanglian Maidong Decoction | Radix Ophiopogonis (Ophiopogon japonicus (Thunb.) Ker-Gawl.) (Mai Dong), Notoginseng Radix et rhizome (San Qi), Coptidis Rhizoma (Huang Lian), |
| Huangqi Guizhi Wuwu decoction | Radix Astragali (Astragalus membranaceus (Fisch.) Bunge) (Huang Qi), Cinnamomi Ramulus (Gui Zhi), Paeoniae Radix Alba (Chi Shao), Zingiberis Rhizoma Recens (Sheng Jiang)and Jujubae Fructus (Da Zao) |
| Huangqi decoction | Radix Astragali (Astragalus membranaceus (Fisch.) Bunge) (Huang Qi), Radix Glycyrrhizae (Glycyrrhiza uralensis Fisch.) (Gan Cao) |
| Qidan junzhi decoction | Radix Astragali (Astragalus membranaceus (Fisch.) Bunge) (Huang Qi), Salviae miltiorrhizae Radix et Rhizoma (Dan Shen)，rhubarb (Radix Rhei Et Rhizome), leech saliva (Shui Zhi) and so on. |
| Danshenyin | Salvia miltiorriza Bunge (Dan Shen), Santalum album (S. album) (Tan Xiang), Amomum villosum Lour. (A. villosum) (Sha Ren) |
| Danggui Buxue Decoction | Radix Astragali (Astragalus membranaceus (Fisch.) Bunge) (Huang Qi), Angelica sinensis (Dang Gui) |
